# English Enhancing Exercises: An effective strategy to upgrade and enhance the English proficiency of medical students in non-English speaking countries

**DOI:** 10.12669/pjms.41.7.12632

**Published:** 2025-07

**Authors:** Nadeem Alam Zubairi

**Affiliations:** 1 Dr. Nadeem Alam Zubairi, Department of Pediatrics, Faculty of Medicine in Rabigh, King Abdulaziz University, Saudi Arabia

**Keywords:** English medium instruction, Language barriers, Medical Education, Medical terminologies

## Abstract

English as a medium of Instruction (EMI) has been adopted by the majority of non-native English-speaking countries for teaching science subjects. Medicine is no exception. The decision is based on enormous associated advantages both during the undergraduate period and in professional life as a doctor. However, the linguistic barrier does impose numerous deficiencies related to proficiency in English. This, if not corrected, leads to suboptimal learning and affects the career and output. Formal and dense measures have been suggested but are against the cognitive load on a medical student. English Enhancing Exercises (EEE) is an easy-to-do student-friendly approach, that gradually and spirally helps out without any additional burden.

## BACKGROUND

In today’s globalized world, the English language has become the primary language of communication in all scientific fields, including medical science. When adopted by non-English native countries, this is often termed English medium of instruction (EMI) and defined as “The use of the English language to teach academic subjects other than English itself in countries or jurisdictions where the first language of the majority of the population is not English”.^1^ In medical science, not only has it been accepted as the medium of teaching, but all major medical books, journals, conferences, training courses, and workshops, as well as examinations, are conducted in English. Optimal learning and output are thus also related to one’s command of English. Similarly, getting benefits from global research or publication of your research is linked with the ability to comprehend and express in English, though other research-related difficulties do exist as well.^2^ There are few, who advocate the use of local language as the medium of learning medicine^3^, but the practice in most of the concerned countries is otherwise, based on the elements mentioned above. To remain connected with the world and progress, English remains a must. This is comparable with the global necessity of the use of English, general and specific, between pilots and air traffic controllers, termed Aviation English.

### Concerns:

There are many issues that medical students face during their years of study.^4^ The linguistic barrier is one as pointed out earlier. Although many undergraduate medical students in non-native English-speaking countries have an excellent command of English writing and expression, still a huge number of them lack the minimum required proficiency. This is mainly due to the basic schooling background. Students from rural setups and non-private schools join the medical colleges with this handicap. As mentioned, it impedes their optimal outcome despite not lacking in I.Q. and effort. This is unfair and should be addressed. Students are using variable techniques to cope with the issue even to the extent of translating the whole topic or available/given material in the local language.^5^ Such efforts are random, time-consuming, and with little long-term benefits. Related subjectivity in exams has been reduced through the use of MCQs, but this too is also counter-productive at times as over-reliance on MCQs has dented English writing skills. Inadequate abilities to understand, write, and express in English leads to poor grades, loss of confidence, anxiety, and lesser attainment of knowledge/skills in college days.^6^ Later, it affects employment, participation in research/conferences, and keeping updated with global progress.^7^ Despite all the difficulties, studies have shown that an overwhelming majority of students are in favor of being taught in English, recognizing the importance and global connection.^8^

### Identification of problem areas where improvement is needed:

Experience has revealed that non-English speaking students have deficiencies in the aspects mentioned below which are affecting them adversely as discussed earlier. The vocabulary of general English is limited. Medicine, in addition, has its specific terminologies and genres. Most students are lacking in both. Learning Medical terms is a process that comes with time and exposure but students may often not understand some common English words, compromising their understanding of the line/talk and resulting in poor understanding and loss of time as well.^9^ Spellings of both English words and medical terms are in general very poor and the reader sometimes even can’t understand what the student has written. This is a major concern for postgraduate years and reflects badly on the individual and the institution, despite him/her being competent in knowledge and skills. Basic grammar mistakes are common, like not starting sentences with a capital letter, what tense to use with ‘did’, etc. Writing skills as a result of all these are compromised. Written or typed medical histories and PowerPoint slides are often substandard. Copy-paste trend is a shortcut that brings problems in years to come. Spoken English however is much better though few students can’t read their self-created slides with fluency


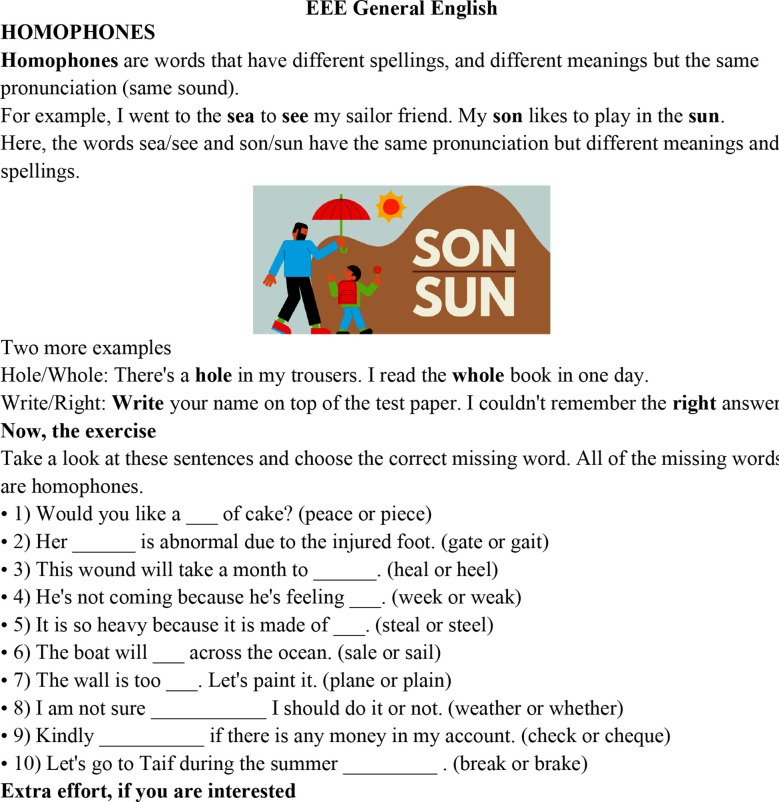



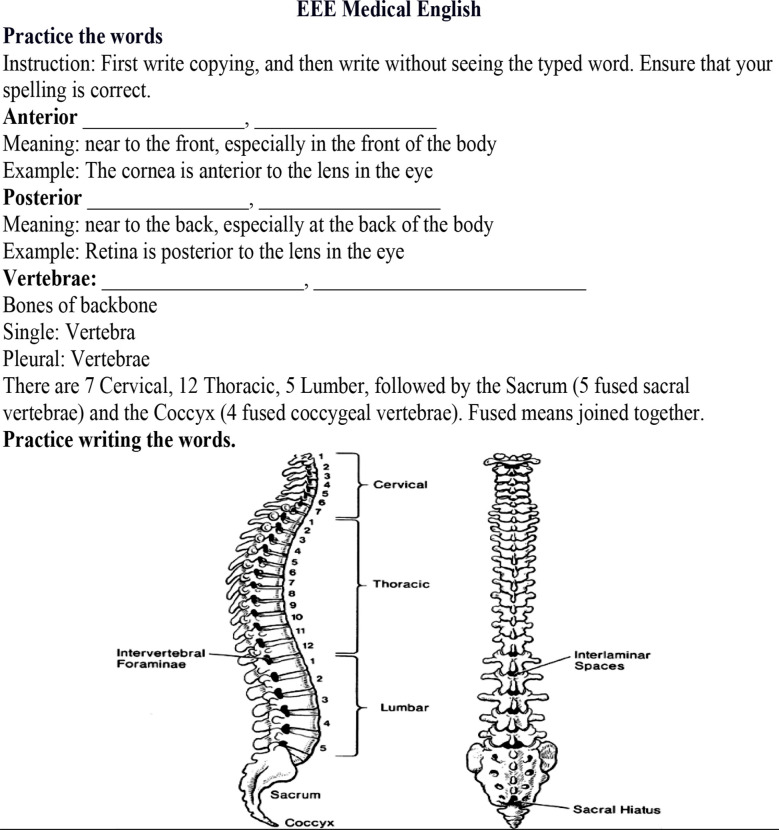


### The Way-out:

Although the challenge of acquiring proficiency in English for better professional outcomes is an additional one for non-English speaking students in all fields, this is even more for medical students due to the extra load of learning in medicine. Very robust and heavy programs have been suggested by many^10^, but these may be counter-productive due to existing cognitive load. Similarly, a bi-lingual teaching approach has been suggested 11 but appears to be cumbersome and less practical. The same is true for adopting peer-assisted teaching of medical English skills to non-native English-speaking medical students, as recommended by a few 12. Balancing the ongoing load on a medical student and the necessity for him/her to be proficient in English, a solution is through east-to-do and easy-to-check **“English Enhancing Exercises” (EEE)**

- These exercises are not a part of the curriculum; rather these are a co-curricular activity extending to three years (1st to 3rd year) with minimal effort or pressure on students. They will learn with enjoyment and ease. The first three years have been intentionally chosen as the mentioned difficulties and its impact is maximum in the initial years in medical college 13. However, if not rectified in these years, it carries its negative influence to final years in medical college and professional life as doctors
- Every month two exercises are posted to the students through a common WhatsApp group. One exercise is related to General English and the other is from Medical English It takes a few minutes for the student to do these. The “key” of the given exercises, where needed, is then posted after three days for self-check**.** The process is taken forward gradually and spirally, bringing improvement, clarity, and confidence in the students.
- ***General English*** exercises cover common grammar mistakes, use of verbs, tenses, adjectives, adverbs, conjunctions, homophones, homonyms, sentence structure, etc. These are given in attractive forms so that learning comes with ease and fun and not as a burden. Self-check keys too are constructed with illustrations and easy-to-grasp techniques. (Example Attached).
- ***Medical English*** is primarily considered as a separate entity as it encompasses unique medical terminologies besides professional medical genres, needed to understand medicine and its subsequent use, like in writing reports. Researchers have suggested the formal inclusion of techniques like the “vocabulary notebook” and “word list”, in the curriculum under the ESP (English for Special Purpose) course 14. As mentioned earlier, all extra burdens imposed formally can be counterproductive. Our approach is to go simple in an easy way without burdening students. Students are encouraged to do two tasks. They are asked to pick five words for each presentation/topic and write it down three times, ensuring correct spelling. Secondly, they are given monthly exercises to augment their understanding of medical terms and their spellings. (Example Attached).
- In addition, the process is supplemented by occasional attractive learning tips, vocabulary-enhancing applications, summary-making drills, articles to read, related videos, and links. All these are optional without compulsion
- Students are encouraged to appear in IELTS during the break between 3^rd^ and 4^th^ year

### Limitations:

The approach has the limitation that the participation is entirely up to the students. Secondly, it cannot lead to peer interaction. These limitations were weighed against the advantages of extreme ease, no time bounding, and a gradual improvement spanning over three years

## CONCLUSION

The importance of English for medical students and doctors cannot be overstated. As the majority of the world’s population is not native English speakers, medical students in such countries have variable proficiency in English, which challenges their learning and output, including in the field of medical research. English Enhancing Exercises Plan is a student-friendly solution to boost English proficiency in medical students of such countries
